# Efficacy of Natural Deep Eutectic Solvents for Extraction of Hydrophilic and Lipophilic Compounds from *Fucus vesiculosus*

**DOI:** 10.3390/molecules26144198

**Published:** 2021-07-10

**Authors:** Ekaterina D. Obluchinskaya, Olga N. Pozharitskaya, Lyubov V. Zakharova, Anna V. Daurtseva, Elena V. Flisyuk, Alexander N. Shikov

**Affiliations:** 1 Murmansk Marine Biological Institute of the Russian Academy of Sciences (MMBI RAS), Vladimirskaya, 17, 183010 Murmansk, Russia; obluchinskaya@mmbi.info (E.D.O.); or pozharitskaya@mmbi.info (O.N.P.); zakharova@mmbi.info (L.V.Z.); tkach@mmbi.info (A.V.D.); 2 Department of Technology of Pharmaceutical Formulations, St. Petersburg State Chemical Pharmaceutical University, Prof. Popov, 14, 197376 Saint-Petersburg, Russia; elena.flisyuk@pharminnotech.com

**Keywords:** ascorbic acid, fucoxanthin, phlorotannins, antioxidant, brown seaweeds, synergy, storage stability

## Abstract

The impact of the composition of natural deep eutectic solvents (NADES) and extraction conditions on the simultaneous extraction of hydrophilic ascorbic acid (AA), phlorotannins (TPhC), and lipophilic fucoxanthin (FX) from *Fucus vesiculosus* was investigated for the first time. In biological tests, the NADES extracts showed the promising ability to scavenge DPPH radicals. A positive correlation was observed between DPPH scavenging activity and AA, TPhC, and FX contents. We calculate the synergistic effect of antioxidants extracted by NADES from *F. vesiculosus* based on the mixture effect (ME). The addition of 30% water to the NADES and the prolongation of sonication time from 20 min up to 60 min were favorable for the ME. The ME for extracts with the NADES was increased by two folds (ME > 2). In contrast, conventional extraction by maceration with steering at 60 °C does not lead to the synergistic effect (ME = 1). It is notable that the NADES provides high stability and preserves the antioxidant activity of the extracts from *F. vesiculosus* during storage.

## 1. Introduction

The development of the environmentally friendly and efficient extraction of biologically active compounds from phytobiomass is a modern trend in the pharmaceutical, cosmetical, and food industries. The natural deep eutectic solvents (NADES) are a promising alternative to conventional organic solvents and gained popularity because they are green, non-toxic, biodegradable, and recyclable [1,2]. The NADES were proposed by a group of scientists who discovered a third liquid phase in plants, which has a phenomenal dissolving power for small molecules and biopolymers with low or non-water solubility [3]. Such solvents consist of the metabolites of living cells: sugars, organic acids, ammonium, and phosphonium salts, etc. The solubility of the target compounds in the NADES significantly increases due to the formation of hydrogen bonds with solutes [4,5].

To be predominantly polar liquids, NADES have been used for the extraction of alkaloids [6], anthocyanins [7,8,9], glycosides of phenyletanes and phenylpropanoids [10], and polysaccharides [11]. NADES could be customized for the extraction of less polar aglycones of flavonoids [12], anthraquinones [13], astaxanthin [14], curcumin [15], glycyrrhizic acid [16], iridoids [17], and steroidal saponins [18]. Hydrophobic terpenes and fatty acid-based NADES allowed for the isolation of carotenoids, free fatty acids, and pheophytin [19]. Recently, a biphasic system based on deep eutectic solvents was proposed for the simultaneous extraction of high polarity chlorogenic acid, quercetin, anthocyanidins, and a low polarity compound artemisinin from Artemisia [20]. Several papers report the application of NADES for the recovery of bioactive compounds from seaweeds [19,21,22,23,24].

Brown seaweeds, particularly, *Fucus vesiculosus*, are rich sources of biologically active metabolites: amino acids [25], fucoxanthin and other carotenoids [26], chlorophylls [27], fucoidan [28,29,30], polyunsaturated fatty acids [31,32], and polyphenols [33,34], etc.

Brown seaweeds, compared with other seaweeds, contain higher levels of polyphenols, which shows potent antioxidant activity. *F. vesiculosus* polyphenols are represented mainly by very hydrophilic phlorotannins-polymerized phloroglucinol (1,3,5-trihydroxybenzene) units [25,35,36]. Another hydrophilic compound of interest in brown seaweeds is ascorbic acid. It was estimated that the daily consumption of 100 g of seaweed covers 2/3 of the recommended doses of ascorbic acid for humans [37]. One of the main lipophilic compounds produced by brown seaweeds is carotenoid fucoxanthin, which is responsible for algae color [38]. Fucoxanthin is a well-established antioxidant according to in vitro and in vivo investigations [39,40].

The antioxidant effects of seaweed extracts have been confirmed both in vitro and in vivo [36,41,42,43]. Epidemiological observations provide evidence that dietary supplementation with seaweeds reduces the risk of numerous diseases associated with oxidative stress [44,45,46]. Therefore, we consider using the antioxidant test as an indicator for the biological activity of novel extracts from *F. vesiculosus* obtained by the application of NADES. Due to peculiar characteristics, including chemical complexity, susceptibility to oxidation, and specific polarity, the simultaneous extraction of hydrophilic and lipophilic compounds is a challenging process.

In this study, NADES were tuned to simultaneously extract hydrophilic and lipophilic bioactive compounds from *F. vesiculosus*. The efficacy of extraction was evaluated by the analysis of the content of ascorbic acid, fucoxanthin, and phlorotannins. The biological activity of the extracts was monitored using an antioxidant activity test. Additionally, the impact of some compounds on antioxidant activity and the ability of NADES for the stabilization of extracts was studied.

## 2. Results and Discussion

### 2.1. NADES Tuning

The widespread use of toxic solvents in chemical, pharmaceutical, and other industries places a heavy burden on the environment. The green extraction of biologically active compounds from phytobiomass with NADES is essential for rational environmental management [47,48]. NADES, mainly synthesized from polar components, are well studied for the recovery of hydrophilic compounds. In this study, we aimed to extract both hydrophilic and lipophilic compounds from *F. vesiculosus*. Based on literature data and the results of our preliminary experiments we have prepared the two most promising NADES (Table 1).

**Figure 1:**
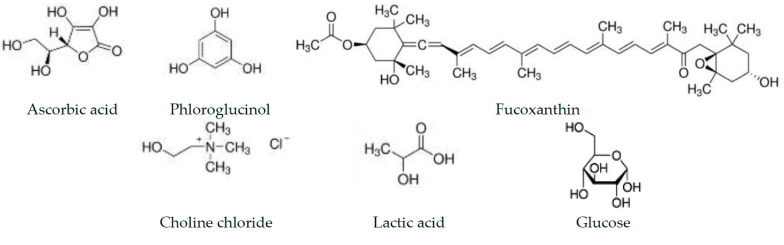
The structures of ascorbic acid, phloroglucinol, fucoxanthin, choline chloride, lactic acid, and glucose.

Although advanced extraction techniques, such as pressurized liquid extraction [49], supercritical fluid extraction [50], and microwave-assisted extraction [51] have been implemented, ultrasound-assisted extraction (UAE) is the most commonly employed method for the recovery of biologically active compounds from brown seaweeds [23,43]. Whereas percolation (PE) is recognized as an exhaustive extraction method [52]. Ethanol has been recommended as a suitable solvent for extraction of ascorbic acid [53], phlorotannins [42,51], and fucoxanthin [54,55]. The structures of the analyzed compounds and components of NADES are presented in Figure 1. Typical chromatograms of reference compounds and NADES extracts are available in Supplementary materials (Figures S1–S7).

The efficacy of the extraction of hydrophilic and lipophilic compounds from *F. vesiculosus* with NADES using UAE was compared with that of ethanol using PE (Table 2).

**Figure 2:**
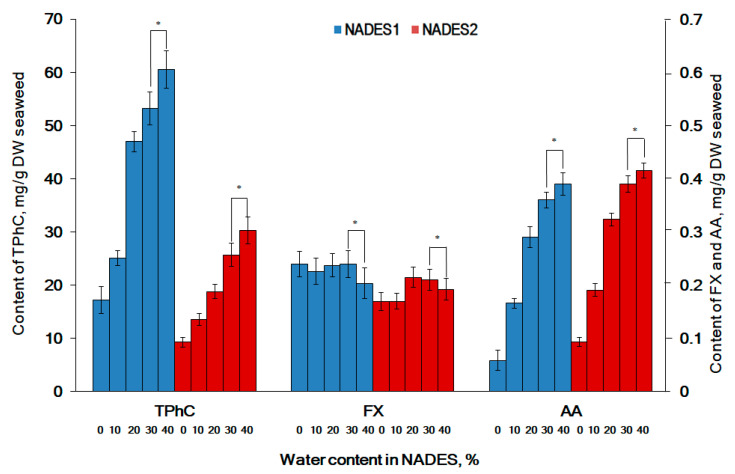
The effect of water content in NADES on the extraction efficacy of total phlorotannins (TPhC), fucoxanthin (FX), and ascorbic acid (AA) from *F. vesiculosus*. All experiments were performed using UAE at a 1:10 (*w*/*v*) seaweed to solvent ratio at 25 °C for 20 min. Water content 0, 10, 20, 30, and 40% (*v*/*v*). * The difference is not statistically significant, *p* > 0.05.

PE provided the maximal recovery of all hydrophilic and lipophilic compounds from seaweeds (Table 1). NADES1 has more potential for recovery of TPhC and FX when compared with NADES2. While NADES2 was more suitable for the extraction of AA (Table 2). The addition of water to NADES led to a decrease in the viscosity and in the surface tension of the solvents, which had a positive impact on the mass transfer from the seaweed cells into the extract [4]. The water (10–40%, *w*/*w*) was added to both NADES to facilitate the efficacy of extraction (Figure 2).

The extraction yields of the hydrophilic ascorbic acid and phlorotannins significantly raised as the water content in the NADES increased. The compounds rich in hydroxyl groups like AA and TPhC are good hydrogen donors and preferably form bonds with hydrogen bond acceptors like choline chloride [23,56]. Although recently, AA has been introduced for the preparation of NADES [56,57], to the best of our knowledge, we report the extraction of ascorbic acid from seaweeds with NADES for the first time. Carotenoid FX is practically insoluble in water. Thus, the addition of water to both NADES does not have a statistically significant effect on the recovery of FX (Figure 2). Organic toxic solvents are commonly used for FX extraction from seaweeds [58]. Recently, Roy et al. (2021) reported on the extraction of another carotenoid astaxanthin from a marine species with NADES [59]. We have not found information about the recovery of FX by NADES in the available literature. This is the first time NADES are suggested for the extraction of fucoxanthin from *F. vesiculosus*.

The addition of water to both NADES, up to 30%, significantly improved the extraction yield of hydrophilic compounds. While an increase of water up to 40% had not resulted in a statistically significant increase of extracted compounds (Figure 2). This is in line with other authors who recommend a maximum limit of 30% of water in NADES [60,61,62]. Therefore, for subsequent experiments, we consider the addition of 30% water to both NADES.

### 2.2. Extraction Conditions Tuning

The duration of the extraction significantly affects the performance and selectivity of the recovery of active principles from seaweeds [62,63]. We observed that the extension of the extraction time leads to an increase in the yield of tested compounds (Figure 2). The most promising increase was observed for FX (up to 89.5–91.3%, when compared to extraction by PE) (Table 1). This is consistent with the literature data that indicates a positive effect of the extraction time on the yield of FX from seaweeds [54,55]. Notably, TPhC values after 60 min sonication with both NADES (Figure 2) were equal to the efficacy of PE (Table 1). This could be explained by the more complete interaction of NADES via numerous hydrogen bonds with the hydroxyl groups of phlorotannins. This promotes better solubilization of phlorotannins after prolonged sonication [64]. Additionally, polyphenols interact with NADES to form polymers over the longer extraction period [65].

**Figure 3:**
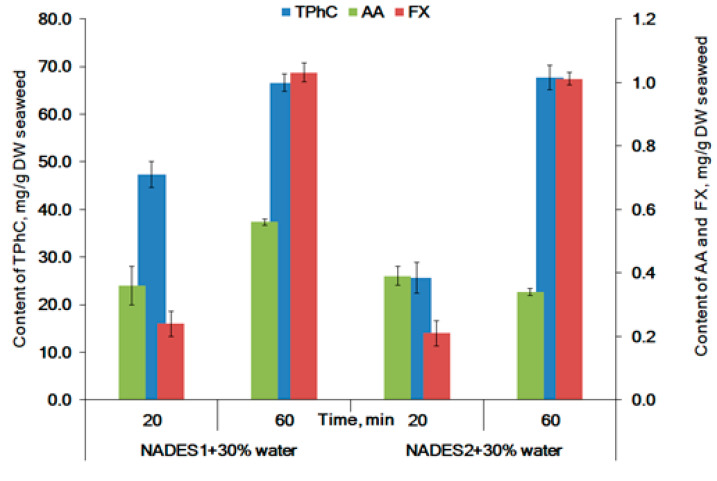
The effect of extraction time on the recovery of ascorbic acid (AA), total phlorotannins (TPhC), and fucoxanthin (FX) from *F. vesiculosus* by UAE at a 1:10 (*w*/*v*) seaweed to solvent ratio at 25 °C.

Prolonged sonication for 60 min resulted in an increase of AA extraction by 55%, compared with 20 min sonication in the case of NADES1. The sonication for 60 min was not favorable for AA extraction by NADES2 (Figure 3). Ultrasonic waves generate intensive cavitation in the extraction medium, resulting in the formation and explosion of cavitation bubbles [66]. These generate mechanical shear forces which destroyed the seaweed cell, thereby facilitating the release of active compounds into the solvent phase. Furthermore, acoustic streaming causes the mixing effect, which enhances the contact between solvents and active compounds and increases extraction performance [67]. Otherwise, the intensive mixing and formation of bubbles resulted in saturation of the extraction media with air. It was shown that the air’s oxygen heavily induced the degradation of AA in juices after 60 min of stirring at 25 °C [68]. All wave phenomena during UAE were attenuated in viscous media. Due to the higher water content, NADES2 has less viscosity when compared to NADES1. The lower AA content in NADES2 could be associated with its degradation during the 60 min of sonication in our experiment.

**Figure 4:**
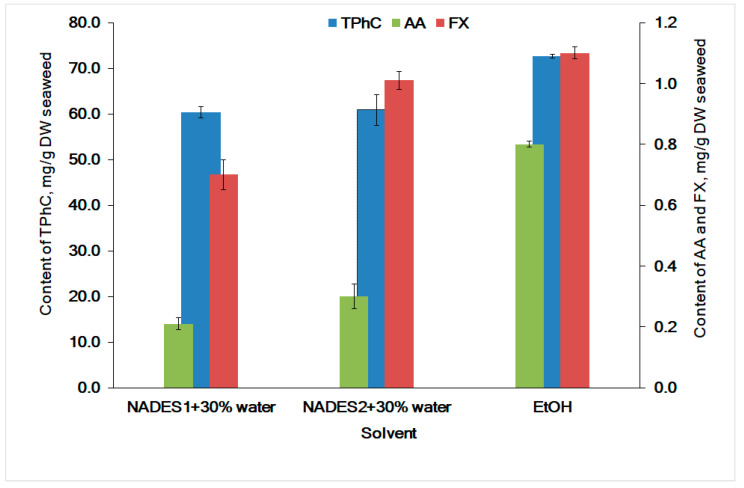
Comparison of extraction efficacy of ascorbic acid (AA), total phlorotannins (TPhC), and fucoxanthin (FX) from *F. vesiculosus* with NADES (CE at 60 °C) and with EtOH by exhaustive extraction (PE, 25 °C).

It is believed that UAE with NADES as extraction solvents, due to their high viscosity, is less efficient than the conventional method of extraction (CE) [69]. Maceration via stirring is CE for the recovery of biologically active compounds from brown seaweeds [33,70]. NADES are viscous substances. Heating decreases viscosity [4] and boosts the convection and mass transfer from the seaweed matrix [71]. Further, we compared the compounds’ yields extracted with ethanol (using PE at 25 °C), NADES (UAE-60 min, 25 °C), and NADES (CE at 60 °C) from the *F. vesiculosus* material. The heating and dilution of NADES with 30% of water (*w*/*w*) promotes a significant increase in the recovery of all compounds by NADES (Figure 4). The most promising results were obtained for NADES2. The yield of AA, TPhC, and FX was increased by 3.2, 6.5, and 5.9 folds, respectively, when compared to UAE extraction at 25 °C for 20 min (Table 2). Whereas the yields of TPhC and FX extracted with NADES (CE, at 60 °C; Figure 4) was equal to those of with NADES (UAE-60 min, at 25 °C; Figure 3). However, the recovery of AA in NADES using CE (at 60 °C) was lower than that of UAE-60 min (at 25 °C). This could be due to the possible degradation of AA at intensive stirring and heating. We consider that the UAE for 60 min at 25 °C is preferable for greater recovery of all lipophilic and hydrophilic compounds from *F. vesiculosus.*

### 2.3. Antioxidant Activity

**Figure 5:**
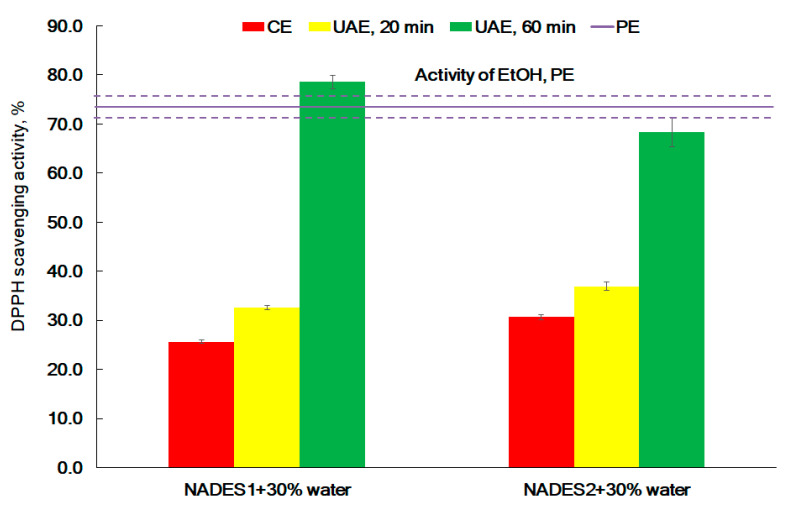
The effect of the extraction method (CE (60 °C), UAE-20 min (25 °C), UAE-60 min (25 °C), and PE (25 °C)) on the antiradical activity of the NADES extracts from *F. vesiculosus*.

DPPH assay is a well-accepted method for the analysis of the antioxidant activity of seaweed extracts [36,39,42,43]. The DPPH assay results are demonstrated in Figure 5. The data indicate that the method and conditions of extraction play a key role in the antioxidant activity of extracts. The lowest activity was observed for extracts received by the CE method. DPPH scavenging activity of extracts obtained by the UAE significantly rises with the extraction time. The extension of the extraction time, up to 60 min, resulted in the increase of activity by 2.4 folds for the NADES1 extract and 2.2 folds for the NADES2 extract when compared to 20 min UAE. The NADES1 extract, prepared by UAE for 60 min, showed 78.5 ± 1.4% DPPH scavenging (Figure 5). This activity was significantly higher than the activity of the PE extract. The experimental data evidenced that NADES with 30 wt% water shows excellent ability for the extraction of compounds from seaweeds with potent antioxidant activity. This corroborates with Ummat et al. (2020), who reported that, compared with CE, ultrasound facilitated the release of polyphenols from the seaweed, which leads to increased DPPH scavenging activity of UAE extracts [43].

Pearson’s correlation analysis evidenced that all hydrophilic and lipophilic compounds observed in this study handle the antioxidant activity. A positive correlation was observed between DPPH scavenging activity and AA (0.76, *p* < 0.0000), as well as TPhC (0.46, *p* < 0.02) and FX contents (0.55, *p* < 0.006). The correlation was also found between AA and TPhC (0.87, *p* < 0.0000), while there was no significant correlation with TPhC and FX, indicating that some individual compounds or fractions could contribute more than others to the antioxidant properties of the *F. vesiculosus* extracts. A similar positive correlation between antioxidant activity and FX and TPhC from seaweeds was observed by other authors [41]. The hydroxyl group in AA and TPhC are responsible for the potent antioxidant activities [43,53]. While the activity of FX is associated with conjugated double bonds and the presence of the peroxide group and allenic bond in the terminal rings of FX [39].

### 2.4. Determination of the Mixture Effect

The natural crude extracts represent a mixture of different compounds. The discovery of the impact of individual compounds/fractions on the antioxidant activities of natural extracts is challenging [36,72]. The mixture effect (ME) is one of the common approaches for expressing the synergistic or antagonistic effects occurring between pairs of antioxidants in a mixture [72,73]. To investigate ME, solutions of AA and phloroglucinol and their mixture were prepared and their ability to scavenge DPPH radicals was studied. Based on the data obtained for the individual solutions and extracts (NADES1, NADES2, and EtOH), the DPPH scavenging activity was calculated after 30 min. Mathematically, ME > 1 evidence regards the synergistic effect between antioxidants, whereas ME < 1 indicates antagonism. In the case of ME = 1, neither a synergistic nor an antagonistic effect exists.

The antioxidant activity for a model mixture of AA and phloroglucinol showed that they have a strong synergistic effect on the reaction with the DPPH radical (ME = 2.99). The PE extract had a pronounced synergistic effect (ME 2.03). In the experiments with UAE, the extension of the sonication time from 20 min up to 60 min was favorable for ME. The ME for the extracts with NADES1 and NADES 2 increased from 0.99 to 1.96 and from 1.00 to 2.27, respectively. In contrast, CE at 60 °C does not lead to the synergistic effect (ME 0.94 and 1.04 for NADES1 and NADES2, respectively). The increased AA and FX content in the NADES2 extract compared to the NADES1 extract (Figure 4) can explain the slightly higher ME for NADES2. The UAE extracts were more beneficial due to the higher concentration of AA and FX in these extracts (Figure 3) compared to CE (Figure 4) and potent antioxidant activities (Figure 5). Although the content of FX in seaweed extracts is lower than the content of TPhC, FX contributes to the antioxidant activity as well [74]. The synergy of antioxidants from seaweeds has been claimed in several papers [36,74,75]. To the best of our knowledge, the synergistic effect for antioxidants extracted by NADES from seaweeds is calculated in this article for the first time.

### 2.5. Storage Stability

**Figure 6:**
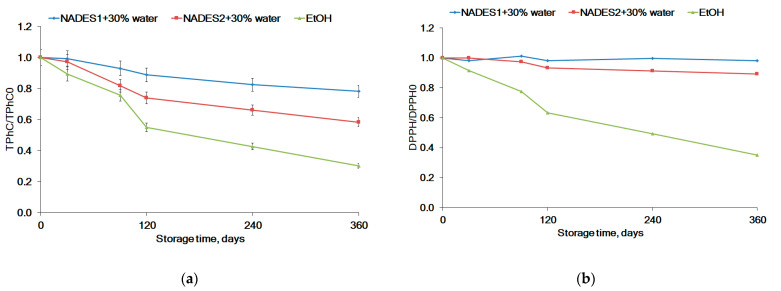
(**a**) The dynamic of the total phlorotannins content (TPhC) and (**b**) DPPH scavenging activity during the storage of *F. vesiculosus* extracts at 25 °C in a dark place for 360 days.

NADES are not only green solvents for natural compounds, but also enhance their stability [62,69]. Phlorotannins were the most abundant metabolite in the studied extracts of *F. vesiculosus*. Therefore, we have monitored the effects of solvents and storage time on the stability of phlorotannins in NADES and EtOH extracts. Both the content of TPhC in the extracts and the appropriate antiradical activity were determined during storage at 25 °C in a dark place for 360 days. The results showed that after 30 days of storage the content of TPhC in EtOH began to decline considerably faster compared with the TPhC in both NADES (Figure 6a). NADES1 enables greater stability of the phlorotannins, while about 70% of the phlorotannins degraded in EtOH after 360 days of the experiment. The stability of TPhC in the extracts (Figure 6a) was correlated with the antioxidant activity (Figure 6b).

Our results are in agreement with literature data in which NADES provides better stability for carthamin [76], anthocyanins [69], and curcumin [15]. The stabilization ability of NADES may have a direct relationship to their viscosity [76]. The total water content in NADES1 is less than in NADES2. The water content in NADES1 is 30% (*w*/*w*), while NADES2 contained three moles of water after preparation (Table 1) and 30% water (*w*/*w*) was added after tuning of solvents (Section 2.1). The dilution with water decreases the viscosity of NADES2 compared to NADES1. The higher viscosity of NADES negatively affects the movement of molecules, allowing a stable interaction between the molecules of the NADES components and the solutes. This leads to a reduction in the contact time for metabolites on the NADES surface with air and consequently resulted in less oxidative degradation [69]. Additionally, oxygen has a lower solubility in NADES than in ethanol or water. The solubility and physical stability of some pharmaceuticals in NADES have also been reported recently [77]. Results of our experiments suggest that NADES enable not only the better stability of active metabolites from *F. vesiculosus*, but also preserve the antioxidant activity of extracts.

## 3. Materials and Methods

### 3.1. Materials and Reagents

Fresh brown seaweeds *Fucus vesiculosus* L. were collected from the coastal region of Zavalishin Bay of the Barents Sea (Russia), 69°11.38′ N, 35°14.78′ E in August 2018. The seaweeds were washed twice in filtered seawater and cleaned from epiphytes by carefully rubbing its surface. The samples were frozen and stored at −25 °C for later extraction. The dry weight was determined from 3 × 5 g of fresh seaweed material, which was dried at 40 °C in the drying oven (UM 200, Memmert GmbH + Co. KG, Germany). The seaweeds were identified by Dr. E. Obluchinskaya and the voucher specimens (No. 8.2018, V.D.Z.) were deposited in the Collection of the Zoobentos Laboratory, Murmansk Marine Biology Institute. L-Lactic acid and D(+)-glucose were from Panreac Química SLU (Barcelona, Spain). The Folin-Ciocalteu reagent, ascorbic acid, phloroglucinol, and 2,2-diphenyl-1-picrylhydrazyl (DPPH) were from Sigma-Aldrich (St. Louis, MO, USA) and the fucoxanthin was from Supelco (Bellefonte, PA, USA). Choline chloride was purchased from Acros Organics (Fair Lawn, NJ, USA). The water was purified via a Milli-Q system (Millipore, Bedford, MA, USA). Other analytical grade chemicals and solvents for extraction and assay were purchased from local chemical suppliers.

### 3.2. NADES Preparation

A heating method [4] was used for the preparation of the NADES. Lactic acid and hydrogen bond donors glucose or choline chloride, at the respective molar ratio, were used [23]. The pre-weighed components (Table 1) were placed in a bottle and heated in a water bath at 50 °C for 60 min with agitation at 700 rpm until a clear liquid was formed. The water-containing NADES were obtained by a dilution of NADES with water based on % weight.

### 3.3. Extraction Conditions

Frozen samples of *F. vesiculosus* were cut into small pieces of about 1−3 mm^2^, thawed for 2 h at room temperature, and then mixed at a ratio of 1:10 (*w*/*v*) with one of the solvent. The ultrasound-assisted extraction (UAE) was performed using a Branson MT-3510 ultrasonic bath (Branson Ultrasonics Corporation, Danbury, CT, USA) at 42 kHz, 130 W for 20 and 60 min. After the sonication, the samples were centrifuged at 3000× *g* for 15 min at 20 °C and a liquid layer was used for future analysis. Conventional extraction (CE) was performed by maceration with magnetic stirring (1 h, 700 rpm) and heating at 60 °C. For percolation extraction (PE), the sample of *F. vesiculosus* was wetted with 96% EtOH for 24 h and then percolated [52] with the same solvent at a ratio of 1:10 at 25 °C. All the extraction procedures were performed in triplicate.

### 3.4. Analysis of Hydrophilic and Lipophilic Compounds

The amount of total phlorotannins content (TPhC) in the extracts was determined according to [78]. Briefly, 100 μL of sample or phloroglucinol was mixed with 2 mL of 2% Na_2_CO_3_ and after 2 min was added 100 μL of Folin-Ciocalteau reagent. The solutions were mixed and incubated for 30 min at room temperature in dark conditions. The absorbance of the reaction was measured at 720 nm using a spectrophotometer Shimadzu UV 1800 (Shimadzu, Kyoto, Japan). The TPhC was expressed as mg phloroglucinol equivalents per gram (mg PGE/g) DW of seaweed.

Fucoxanthin (FX) was analyzed using a reverse-phase high performed liquid chromatography (RP-HPLC) at room temperature [79] with a Shimadzu (Kyoto, Japan) HPLC system comprising of two LC20AD pumps, a DGU-20 A3 degasser, and a SPD.M20 A diode-array detector. Separation was achieved on a 4.6 mm i.d. × 250 mm, 5 µm particle, C18 column (Phenomenex, Torrance, CA, USA) with a SecurityGuard pre-column (2 mm) containing the same adsorbent (Phenomenex). Isocratic elution was performed with acetonitrile-methanol 95:5 (*v*/*v*) as mobile phase at a flow rate of 1 mL/min. The sample injection volume was 20 µL. A Shimadzu LC Solution data-analysis system was used. Chromatograms were registered at 450 nm and the quantification of FX was executed via the calibration curve, with fucoxanthin as a reference. The content of FX was expressed as mg of FX/g DW of seaweed.

Ascorbic acid (AA) was determined by HPLC, as described previously [80], in slight modification. Briefly, 1.0 g of extract was dissolved in 5 mL of water. Isocratic elution was performed with 0.03% aqueous trifluoroacetic acid-methanol 95:5 (*v*/*v*) as mobile phase; the flow rate was 1.0 mL/min. The detection wavelength was 240 nm. Sample volume was 20 μL and each sample was analyzed in triplicate. The calibration curve with AA as a standard was used to calculate the AA in extracts. The content of AA was expressed as mg of AA/g DW.

### 3.5. Antioxidant Activity

The DPPH free radical reacts directly with the antioxidants. The DPPH scavenging activity was analyzed, as previously reported [81]. Briefly, 1 mL of sample or standard was mixed well with 1.5 mL H_2_O and 0.5 mL of 100 µM DPPH methanolic solution in a test tube. AA was used as positive control. The same concentration of methanol and DPPH was used as the control without AA or extract. The reactive solutions were left in darkness at room temperature for 30 min. Then, the absorbance at 517 nm was taken using a UV-Vis spectrophotometer Shimadzu UV 1800 (Shimadzu, Kyoto, Japan). The percentage of antioxidant activity in the different samples was calculated as:

(1) $$\mathrm{DPPH}\;\mathrm{scavenging}\;\mathrm{activity}\;\left(\%\right)=\frac{A_{control}-A_{sample}}{A_{control}}\times 100$$

where A*_control_* is the absorbance of the control and A*_sample_* is the absorbance of the sample.

### 3.6. Determination of Mixture Effect

The mixture effect (ME) of the two antioxidants could be defined as the experimental value, divided by the calculated value, in which the sum of the effects of the two antioxidants is obtained individually [72]. The ME, in the case of the DPPH assay [73], was computed by comparing the experimental DPPH scavenging activity of the extract (“experimental DPPH scav.act.”) with the expected DPPH scavenging activity, calculated by the sum of efficiencies of each compound individually (“calculated DPPH scav.act.”)

(2) $$\mathrm{ME}=\frac{\mathrm{experimental}\;\mathrm{DPPH}\;\mathrm{scav}.\mathrm{act}.}{\mathrm{calculated}\;\mathrm{DPPH}\;\mathrm{scav}.\mathrm{act}.}\;$$

### 3.7. Storage Stability Test

The stability of the NADES1, NADES2, and EtOH extracts was tested. The extracts were stored at 25 °C in the dark and samples of each group were analyzed after 30, 90, 120, 240, and 360 days.

### 3.8. Statistical Analysis

All statistical analyses were performed using STATGRAPHICS Centurion XV software (StatPoint Technologies Inc., USA). The data are expressed as mean ± standard deviation (±SD) and the error bars in the figures indicate the standard deviation. Differences between the means were analyzed via the ANOVA test, followed by the post-hoc Tukey’s test. A significant difference was considered at the level of *p* < 0.05. Pearson’s correlation coefficients were used to establish the relationship between the content of the representative compounds and the antioxidant capacity.

## 4. Conclusions

In this paper, we report for the first time lactic acid: choline chloride and lactic acid: glucose: water-based NADES are suitable for the simultaneous extraction of hydrophilic (ascorbic acid and phlorotannins) and lipophilic (fucoxanthin) compounds from *F. vesiculosus*. The efficacy of UAE for 60 min was, for different compounds, 1.1–2.7 folds higher than the conventional extraction (maceration with stirring at 60 °C). In biological tests, both NADES extracts showed the favorable ability to scavenge DPPH radicals which were equal to the antioxidant activity of EtOH extract. Ascorbic acid, TPhC, and FX significantly contributed to the DPPH scavenging activity of extracts. We calculated the synergistic effect for antioxidants extracted by NADES from *F. vesiculosus* based on the mixture effect for the first time. Notably, NADES, besides their reduced environmental impact, enable high stability for active metabolites from *F. vesiculosus* and preserve the antioxidant activity of extracts. Thus, the results of our experiments highlight the potential of NADES for the recovery of hydrophilic and lipophilic compounds from *F. vesiculosus*.

## Figures and Tables

**Table 1:** Natural deep eutectic solvents (NADES) used for extraction.

NADES Code	Components	Molar Rate	Appearance
NADES1	Lactic acid:Choline chloride	3:1	Viscous transparent colorless liquid
NADES2	Lactic acid:Glucose:H_2_O	5:1:3	Transparent liquid

**Table 2:** The content of hydrophilic-ascorbic acid (AA), total phlorotannins (TPhC), and lipophilic-fucoxanthin (FX) compounds from *Fucus vesiculosus* after UAE and PE (mean ± standard deviation).

Solvent	AA, mg/g DW Seaweed	TPhC, mg/g DW Seaweed	FX, mg/g DW Seaweed
UAE, NADES1	0.058 ± 0.010	17.2 ± 3.7	0.24 ± 0.03
UAE, NADES2	0.093 ± 0.010	9.3 ± 0.9	0.17 ± 0.02
PE, EtOH	0.80 ± 0.01	71.7 ± 0.4	1.10 ± 0.02

UAE (ultrasound-assisted extraction) at a 1:10 (*w*/*v*) seaweed to solvent ratio, 25 °C, 20 min; PE—maceration with EtOH for 24 h, and percolation at a 1:10 (*w*/*v*) seaweed to solvent ratio, 25 °C.

## Data Availability

The data presented in this study are available in supplementary material.

## Supplementary Materials

The following are available online, Figure S1: Typical chromatogram of ascorbic acid (reference), Figure S2: Typical chromatogram of *Fucus vesiculosus* extract obtained with NADES1 (conventional extraction), Figure S3: Typical chromatogram of *Fucus vesiculosus* extract obtained with NADES1 (ultrasound assisted extraction), Figure S4: Typical chromatogram of fucoxanthin (reference), Figure S5: Typical chromatogram of *Fucus vesiculosus* extract obtained with EtOH (percolation), Figure S6: Typical chromatogram of *Fucus vesiculosus* extract obtained with NADES1 (ultrasound assisted extraction), Figure S7. Typical chromatogram of *Fucus vesiculosus* extract obtained with NADES2 (ultrasound assisted extraction).

## Sample Availability

Samples of the extracts are available from the authors.
