# Supplementary material for: Efficacy of Natural Deep Eutectic Solvents for Extraction of Hydrophilic and Lipophilic Compounds from Fucus vesiculosus
Source: Molecules. 2021 Jul 10;26(14):4198. doi: 10.3390/molecules26144198 (PMC8304060; doi:10.3390/molecules26144198)
Supplement: Supplementary file 1 [file molecules-26-04198-s001.zip › molecules-1273161-supplementary.pdf]

Supplementary materials

Efficacy of Natural Deep Eutectic Solvents for Extraction of Hydrophilic and Lipophilic compounds from *Fucus vesiculosus*

Ekaterina D. Obluchinskaya [1], Olga N. Pozharitskaya [1], Lyubov V. Zakharova [1], Anna V. Daurtseva [1], Elena V. Flisyuk [2] and Alexander N. Shikov [1, 2*]

[1] Murmansk Marine Biological Institute of the Russian Academy of Sciences (MMBI RAS), Vladimirskaya, 17, 183010 Murmansk, Russia

[2] Department of Technology of Pharmaceutical Formulations, St. Petersburg State Chemical Pharmaceutical University, Prof. Popov, 14, 197376, Saint-Petersburg, Russia

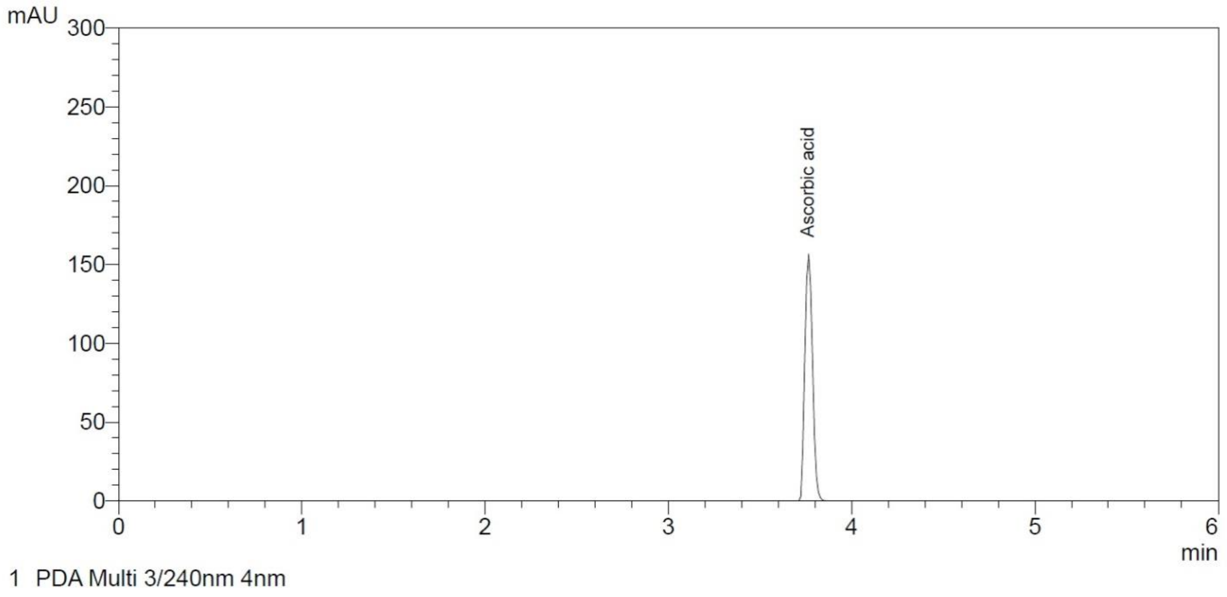

Figure S1. Typical chromatogram of ascorbic acid (reference)

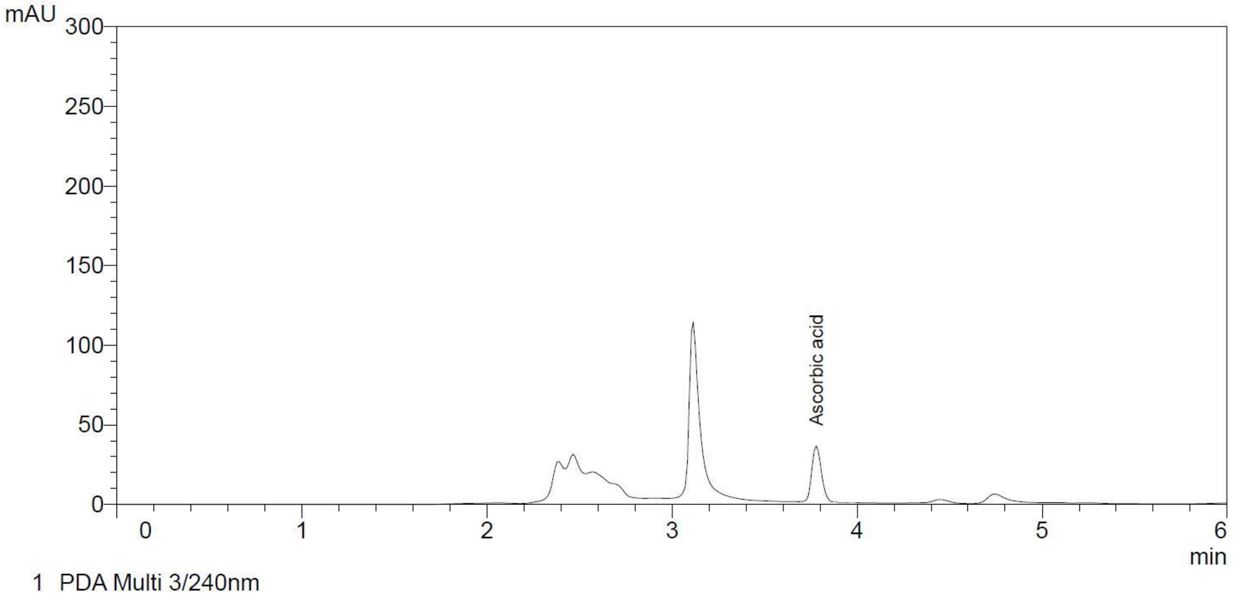

Figure S2. Typical chromatogram of *Fucus vesiculosus* extract obtained with NADES1 (conventional extraction)

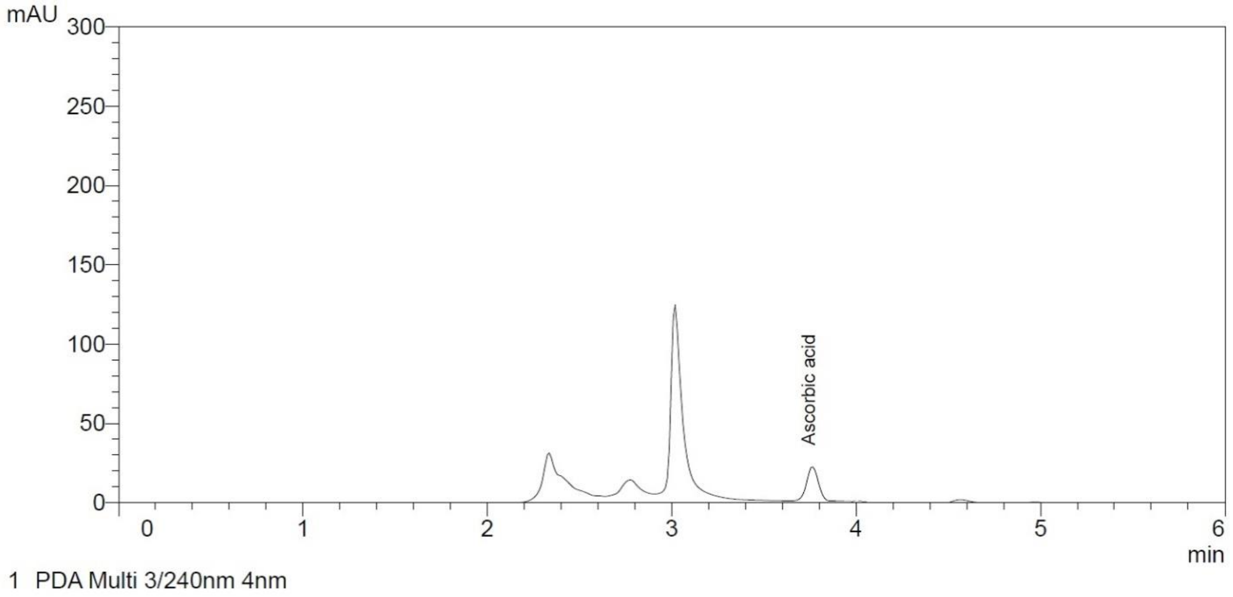

Figure S3. Typical chromatogram of *Fucus vesiculosus* extract obtained with NADES1 (ultrasound assisted extraction)

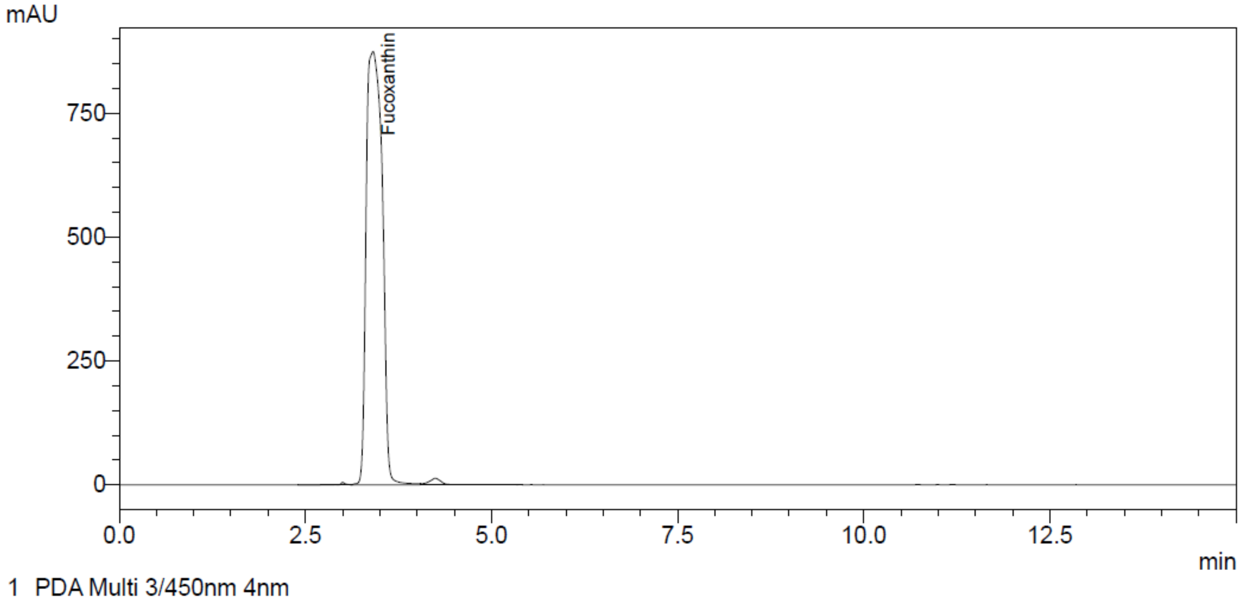

Figure S4. Typical chromatogram of fucoxanthin (reference)

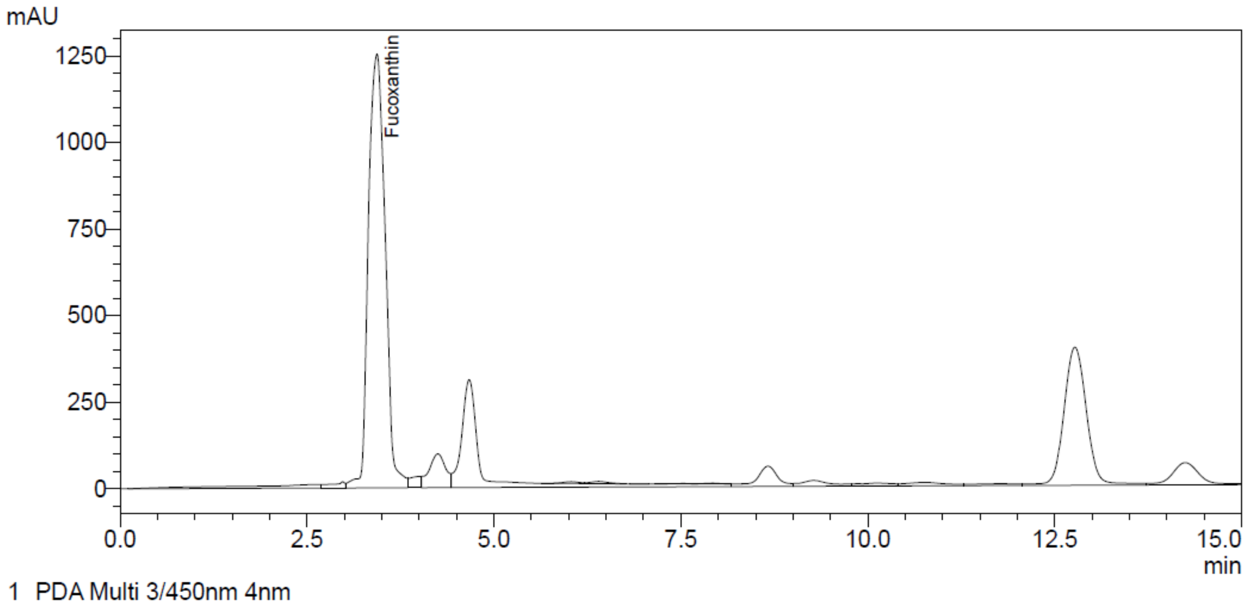

Figure S5. Typical chromatogram of *Fucus vesiculosus* extract obtained with EtOH (percolation)

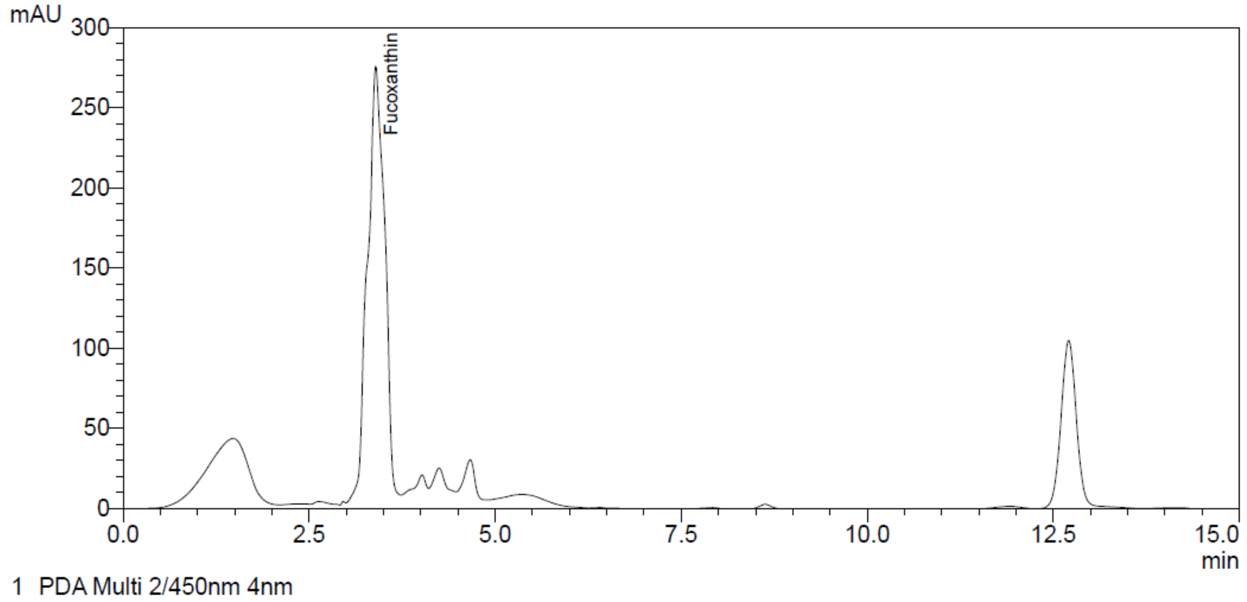

Figure S6. Typical chromatogram of *Fucus vesiculosus* extract obtained with NADES1 (ultrasound assisted extraction)

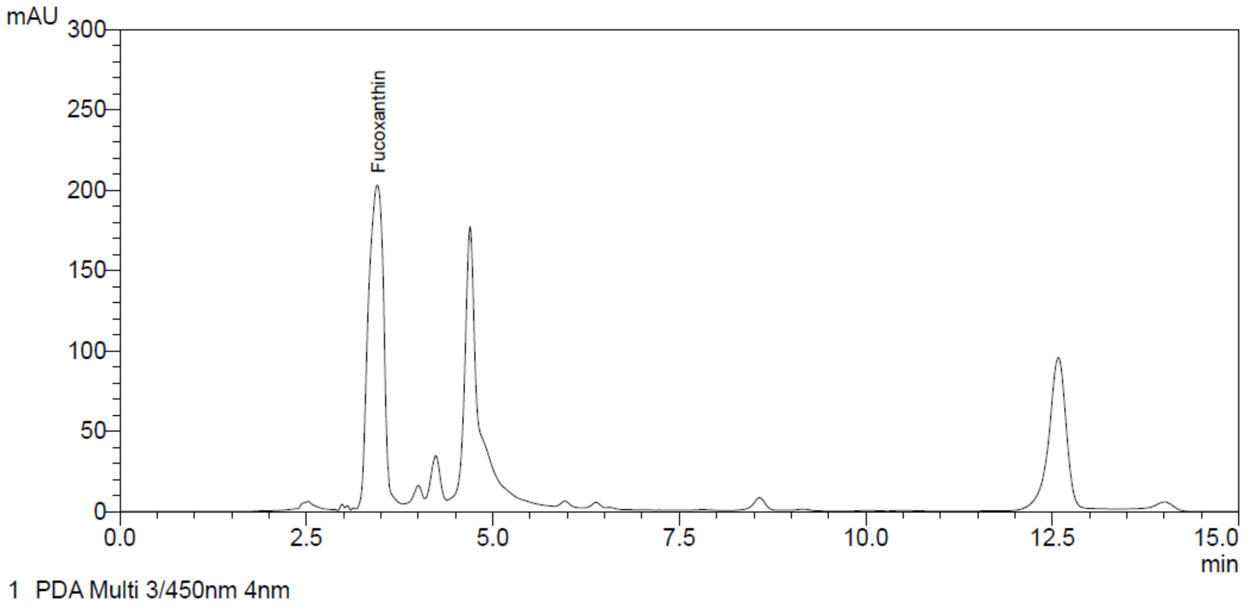

Figure S7. Typical chromatogram of *Fucus vesiculosus* extract obtained with NADES2 (ultrasound assisted extraction)
